# Boosting RSV Immunization Uptake in The Netherlands: (Expectant) Mothers and Healthcare Professionals’ Insights on Different Strategies

**DOI:** 10.3390/vaccines13101051

**Published:** 2025-10-14

**Authors:** Lisanne van Leeuwen, Lisette Harteveld, Lucy Smit, Karlijn Vollebregt, Debby Bogaert, Marlies van Houten

**Affiliations:** 1 Department of Paediatrics, Spaarne Hospital, P.O. Box 417, 2035 RC Haarlem, The Netherlands; 2 Department of Vaccine, Infection and Immunology, Spaarne Hospital, P.O. Box 417, 2035 RC Haarlem, The Netherlands; 3 Willem Alexander Children Hospital, Leiden University Medical Center, P.O. Box 9600, 2300 RC Leiden, The Netherlands; 4 Youth Health Care Centre, Jeugdgezondheidszorg Kennemerland, 1991 JL Velserbroek, The Netherlands; 5 Department of Obstetrics and Gynaecology, Spaarne Hospital, P.O. Box 417, 2035 RC Haarlem, The Netherlands; k.vollebregt@spaarnegasthuis.nl; 6 Department of Paediatric Immunology and Infectious Diseases, Wilhelmina Children’s Hospital and University Medical Centre Utrecht, P.O. Box 85090, 3508 TC Utrecht, The Netherlands; 7 The Centre for Inflammation Research, Institute for Regeneration and Repair, University of Edinburgh, Edinburgh EH16 4UU, UK

**Keywords:** RSV, vaccination, neonatal immunization, prevention

## Abstract

**Background:** Respiratory syncytial virus (RSV) is a major cause of infant respiratory illness, leading to significant hospitalizations. Two preventive strategies exist: maternal vaccination and a long-acting monoclonal antibody for neonates. In The Netherlands, neonatal immunization is planned to start from autumn 2025 onward, contingent on acceptance by parents and healthcare professionals. Maternal vaccination is already available at own costs. Understanding acceptance, perceptions, and barriers is critical for effective implementation. This study explores these factors to inform strategies for optimal uptake. **Methods:** This mixed-method study involved semi-structured online interviews with 21 (expectant) mothers (EMs) and 32 healthcare professionals (HCPs) involved in maternal and neonatal care (e.g., pediatricians, youth doctors/nurses, obstetricians, midwives, and general practitioners) and a quantitative descriptive analysis of factors influencing EM choices. Interviews were transcribed and thematically analyzed. **Results:** Both EMs and HCPs showed strong support for RSV immunization, with a preference for maternal vaccination or a combined approach. Concerns about neonatal injections during the sensitive postpartum period and unfamiliarity with newborn injections (e.g., vitamin K) influenced preferences. EMs noted hesitation about additional pregnancy/postpartum vaccinations, emphasizing the importance of well-timed interventions. HCPs highlighted logistical challenges, such as defining responsibilities, navigating National Immunization Program (NIP) changes, and ensuring readiness. All interviewed individuals value the option to choose between strategies, necessitating informed decision-making and respect for preferences. EMs make their final decision together with their partner, supported by expert information and their personal environment. **Conclusions:** Support for RSV immunization is high, with maternal vaccination preferred, though neonatal immunization is accepted if appropriately timed. Providing clear personalized and consistent information, heightened public awareness of RSV’s impact, respecting individual choices, and offering options are key to maximizing uptake.

## 1. Introduction

The introduction of RSV preventive measures is set to drastically transform the landscape of respiratory infections in early childhood. The burden of RSV infections, characterized by overcrowded pediatric wards and intensive care units during the winter season, may soon become a thing of the past. Two preventive strategies are currently proven to be safe and effective for at least the first 5 to 6 months of life: maternal vaccination (Abrysvo) and a long-acting monoclonal antibody for neonates (Nirsevimab) [1,2,3]. Several countries across Europe, North America, and Australia have either implemented or are in the process of approving one or both RSV protection strategies. In The Netherlands, maternal vaccination is available at own costs and implementation of neonatal immunization in the National Immunization Program (NIP) is planned for autumn 2025. Notably, Spain has reported a substantial decline in RSV cases following the introduction of Nirsevimab, highlighting the potential impact of these measures [4].

Given these significant benefits, widespread acceptance of RSV prevention strategies is anticipated. This is supported by several cross-sectional studies in the UK, Canada and The Netherlands. In the UK, 88% of 1620 pregnant women and mothers with young children indicated willingness to accept maternal vaccination, and 79% would accept neonatal immunization [5]. Similar results were found in Canada, 79% of 723 respondents would accept at least one of the RSV prevention options [6]. Most women preferred maternal vaccination over neonatal immunization in both study populations. A recent study by our group among 1001 pregnant women and their partners showed an 87% acceptance rate for both strategies, again confirming the preference for maternal vaccination, particularly among multiparous women and those intending to breastfeed [7].

Nevertheless, successful implementation of RSV preventive strategies cannot rely on public acceptance alone and several barriers remain. Experiences with the NIP in The Netherlands have shown that implementation success is shaped by multiple interacting factors. These include the accessibility of vaccination services, the clarity and credibility of information provided to parents, trust in healthcare professionals, and effective coordination within the healthcare system [8]. Concerns about expanding immunization programs, limited time for sufficient training of healthcare professionals to offer counselling and rising vaccine hesitancy contribute to declining vaccine trust in The Netherlands, especially post COVID-19 [5,7,9,10]. Research also shows that overly benefit-focused messaging may reduce credibility, especially among groups already sceptical of governmental health advice [9,11]. These findings underscore that even the most promising strategy requires a strong, trust-based implementation approach and need to address not only individual willingness, but also the broader social, emotional, and structural factors that shape vaccine decision.

Effective and equitable RSV prevention in The Netherlands requires insight into the motivations, concerns, and practical challenges of those involved. This study goes beyond our earlier UPTAKE study [7] by providing greater depth and nuance through in-depth interviews and explored pregnant women’s willingness and considerations regarding maternal and/or neonatal immunization, as well as the barriers and facilitators experienced by healthcare professionals. The goal is to inform policies that are both effective and acceptable in practice.

## 2. Methods

A mixed-method cross-sectional study with semi-structured interviews was conducted between July and September 2024 among pregnant women, mothers, and healthcare professionals. This study is approved by the local feasibility committee of the Spaarne Hospital. All participants provided informed consent prior to participation. The COREQ checklist was utilized as a guidance tool for reporting this study (Table 1 and Table S1) [12].

### 2.1. Study Population and Recruitment

The study population consisted of two distinct groups: (Expectant) mothers (EM), and HealthCare Professionals (HCP) who are involved in the care of pregnant women and children in The Netherlands.

The EM group consisted of participants from the UPTAKE study [7], a prior survey-based investigation in pregnant women from our study group, who had indicated their willingness to participate in follow-up interviews. Participants from the UPTAKE study were eligible regardless of whether they were currently pregnant or had already given birth. Inclusion was limited to individuals with sufficient proficiency in the Dutch or English language. Recruitment was conducted by randomly selecting participants from the UPTAKE database, with an emphasis on achieving a balanced sample of women who were likely in favour of RSV prevention methods and those who were likely opposed, based on their previous survey responses. They were asked by email and/or phone to participate.

HCP from six disciplines relevant to the care of pregnant women and children in The Netherlands were also included in the study and consisted of general practitioners, paediatricians, obstetrician, midwives, youth healthcare physicians, and youth healthcare nurses. In The Netherlands, these professionals collaborate to provide comprehensive care, each playing a specific role depending on the stage of pregnancy, the postnatal period, and neonatal or childhood care. General practitioners provide general healthcare for all individuals, including pregnant women, neonates, and children, and refer patients to specialists when needed. Obstetricians specialize in pregnancy and childbirth care, with a particular focus on high-risk pregnancies or complications. In general, they work in the hospital. Midwives oversee uncomplicated pregnancies and deliveries, offering continuous support during pregnancy, labour, and the postpartum period. They also perform the initial health checks for newborns after uncomplicated births. Paediatricians provide care for newborns and children, particularly in cases of medical complications. Youth healthcare physicians and nurses focus on the health and development of children, provide screening and guidance to parents, and administer vaccinations to children according to the NIP. They also educate and vaccinate pregnant women, currently offering the maternal Tdap (pertussis) vaccine. Participants were approached through a combination of professional networks, direct contacts with colleagues, and outreach via professional associations related to the six targeted disciplines. We intentionally sought to achieve a balanced representation across these disciplines, as well as geographical diversity throughout The Netherlands, by inviting professionals working in different regions and serving varied patient populations. Recruitment efforts included personalized invitations and follow-up communications to encourage participation. To minimize bias and potential conflicts of interest, professionals affiliated with the Spaarne Hospital were excluded from the study. This approach was designed to ensure a diverse and representative sample of healthcare professionals involved in maternal and neonatal care.

### 2.2. Data Collection

Data collection consisted of audio- and video-recorded interviews of approximately 30 min, conducted online by two people: LL or LH (both medical doctors and postdoctoral researchers at the Spaarne Hospital) and one of two research nurses (working at the Spaarne Hospital). Interview guides, based on predefined topic lists tailored to the participant group, were used and adjusted during the process if new relevant themes emerged (Table S2). The interview began with open-ended questions, after which participants were encouraged to elaborate. Interviewers explored further into topics introduced by the interviewees and ensured that all key subjects were addressed. Additionally, field notes were taken to capture relevant observations. Participant recruitment continued until thematic saturation was reached, which occurred after the inclusion of 21 EMs and 32 HCPs. Saturation refers to the point at which no new themes or insights emerged from the data, indicating that additional interviews were unlikely to yield further meaningful information. Thematic saturation was assessed informally through weekly team discussions after approximately every five interviews, during which researchers jointly evaluated whether new insights were still emerging. Data analysis was conducted after all interviews were completed.

At the end of the interview with EMs, participants were asked to allocate 100 points among various factors they believed influenced their decision to vaccinate or immunize their child, or for themselves. Participants assigned points, which allowed for an analysis of the key influences on their vaccination decisions.

### 2.3. Data Analysis

The interviews were audio- and video-recorded and automatically transcribed using Microsoft Word. The transcripts were then manually reviewed and corrected as needed by re-listening to the interviews. All transcripts were anonymized to ensure participant confidentiality. To familiarize themselves with the data, the researchers read and re-read the transcripts. Data were initially reviewed through a process of open coding by two investigators independently (LL and LH). Subsequently, the data were analysed thematically. Main themes emerged from existing theory and data collected during the UPTAKE study [7], which had been conducted by the same investigators prior to this study. Themes were finalized during consensus meetings. The data were interpreted and described based on their meaning. Coding disagreements were discussed and resolved collaboratively among the researchers (LL and LH, and if necessary, with a third member of the team MH) to ensure consistency and reliability in the analysis. The qualitative data analysis software MAXQDA Analytics Pro (version 24.4.1) was used to conduct the analysis.

For the characteristics table, numbers with percentages or means with Q1–Q3 were used. To provide an overview of the allocated points by pregnant women and mothers regarding what influences their decision, the percentage of the total points per category was calculated, as well as the absolute distribution of points within each category.

## 3. Results

A total of 35 EMs were asked to participate in this study, of which 21 EMs agreed and were individually interviewed for a median time of 25 min (Q1: 21 min–Q3: 28 min). EMs had a median age of 31 years (Table 2), with nine (42.8%) being pregnant and the remaining 12 (57.1%) having recently given birth. Nearly half (47.6%) were multiparous. Immunization attitudes varied within the group, as previously assessed in the UPTAKE study. In total, 42.9% supported both maternal and neonatal immunization, while 19.0% were likely in favour of one of these or both methods. Another 14.3% were uncertain about maternal vaccination and leaned negative on neonatal immunization. Overall, 23.8% were hesitant or negative about both strategies.

In Table 3, the baseline characteristics of the HCP are presented. A total of 32 HCP were interviewed, with five or six participants from each professional group, of which one interview was conducted jointly with two HCP (a youth healthcare physician and a nurse). The HCP worked across different regions in The Netherlands, with the majority based in the west (34.4%) and south (31.3%). They served diverse populations, including urban, provincial, mixed areas and the Dutch bible belt. The median duration of the interviews with HCP was 29 min (interquartile range: 24–35 min).

The qualitative analysis of all interviews combined identified five main themes: *RSV Awareness & Impact, RSV Protection & Vaccine Hesitancy, The Most Suitable Approach, Timing of Neonatal Immunization*, and *Information*. For illustrative quotations corresponding to each theme, see Table 4. Finally, we discuss the influence on vaccination decisions, explored through a point allocation exercise at the end of the interview.

### 3.1. RSV Awareness & Impact

While all HCPs were familiar with RSV, awareness among EMs varied. Many had heard of it, often through personal or community experiences, or media coverage during the RSV season. Those with direct or indirect experience with RSV were more aware of its severity, particularly due to hospitalizations. Others, lacking such experience, perceived RSV as less concerning.

HCPs, on the other hand, were well-acquainted with RSV and frequently observed the consequences in daily practice. Paediatricians and general practitioners reported increased consultations and hospital admissions during RSV season, straining care capacity. Obstetricians and midwives noted pressure on maternal care due to crowded paediatric wards. Their professional and sometimes personal experiences reinforced the importance of RSV prevention. They also observed varying levels of parental concern, ranging from serious worry to seeing RSV as a mild cold.

### 3.2. RSV Protection & Vaccine Hesitancy

Both HCPs and most EMs acknowledge RSV’s severity and supported the inclusion of a vaccination in the NIP, especially those with personal experience. EMs cited trust in routine vaccination, established research, and a desire to prevent severe illness. HCPs noted limited information about its rollout in the NIP so far and noted that demand remains low, though demand is growing with increased media coverage.

A shared concern was the declining vaccination rate and rising skepticism since the COVID-19 pandemic. HCPs worried that recent NIP expansions may trigger resistance, though they believed young parents are generally receptive to RSV protection, given its perceived seriousness. EMs had more critical attitudes toward vaccines, with questions about safety, ingredients, and testing. The rarity of some vaccine-preventable diseases also reduced perceived urgency. Concerns persisted about potential risks to the (unborn) child and the novelty of RSV vaccines.

Finally, some pro-vaccine EMs expressed concern about unvaccinated children in daycare, citing health risks for vulnerable infants and raising ethical and policy questions.

### 3.3. Timing of Neonatal Immunization

There was no clear consensus among EMs on the ideal timing of neonatal immunization. Some suggested combining it with the heel prick screening to minimize additional procedures, while others preferred delaying it until the first child health clinic visit. The perception that early neonatal injections are uncommon in The Netherlands, where routine vaccinations start at two to three months and vitamin K is given orally, also influenced preferences.

Among HCPs, timing and responsibility were key concerns. To ensure early protection, midwives would need to administer the injection in the first week, as they are the primary caregivers during this period. However, midwives opposed this, citing the vulnerability of the postpartum period and the availability of maternal vaccination as a less disruptive alternative. Several midwives also mentioned the discussion around the vitamin K injection directly after birth. This injection also lacked support within their professional group due to the availability of an effective oral alternative. Because this injection is not part of the standard healthcare offered within The Netherlands, there is a lack of familiarity among young parents, making them more likely to resist immunization directly after birth.

HCPs showed greater acceptance of administering the vaccine toward the end of the first month, primarily for practical reasons. In The Netherlands, all vaccinations fall under the responsibility of youth healthcare services, whose professionals expressed willingness to take on postnatal RSV immunization. However, they typically see newborns between weeks two and four for the first time, and sometimes only online for experienced parents with previous children, making early in-person administration logistically challenging. Obstetricians and pediatricians saw no barriers to vaccinating hospitalized newborns during the first week.

### 3.4. The Most Suitable Approach

Most EMs preferred maternal vaccination if given the choice. However, many also stated that if neonatal immunization becomes the standard, they follow that recommendation. Their primary concern was their child’s protection, which outweighed their personal preference. Their decision was largely guided by the concept of minimal invasiveness for the child and on which option provided the best immunity. Therefore, facilitators for maternal vaccination included avoiding an injection for the baby and ensuring immediate sufficient protection after birth, particularly emphasizing the need for coverage during the RSV season. In contrast, neonatal immunization was seen as beneficial due to its ability to provide protection regardless of seasonal variations. Additionally, EMs emphasized the importance of extensive research and healthcare policies that support routine vaccination regardless the method.

Barriers to maternal vaccination included concerns about timing and potential risks during pregnancy. For neonatal immunization, emotional resistance to injecting a newborn, concerns about vulnerability, and the overwhelming postpartum period were key obstacles. Many EMs noted that their emotional state around childbirth influenced their decision-making.

HCPs were divided on the preferred strategy for RSV protection. Most believed that women would favor maternal vaccination, like the pertussis ‘22-week shot’, which is already considered standard care and offers immediate protection after birth. Ideally, RSV vaccination would be integrated with existing prenatal care. However, some HCPs noted persistent concerns among pregnant women about vaccine safety during pregnancy. Midwives also highlighted logistical and financial barriers, as pregnant women currently receive information from midwives and obstetricians but must visit youth healthcare services themselves to get vaccinated. Some face practical or financial barriers to do so.

Despite these challenges, most HCPs and EMs emphasized the importance of respecting the autonomy of pregnant women. A combination of options or providing a choice between maternal and neonatal immunization was suggested as the most effective approach, as it is expected to result in the highest uptake of RSV protection.

### 3.5. Information

EMs emphasized the need for accurate, balanced, and accessible information to feel well-informed. HCPs also observed growing demand for trustworthy sources, noting that expectant parents increasingly turn to (social) media. There was strong interest in independent, multilingual video content.

EMs preferred information that presents both benefits and risks of vaccination in a clear, rational manner. They stressed the importance of portraying RSV realistically, reassuring parents about possible mild RSV-symptoms and creating awareness of RSV-severity. HCPs agreed, though acknowledged the challenge of remaining neutral when personally convinced of vaccination benefits.

Both groups valued expert input and personal stories and agreed that repeating information across consultations improves understanding. EMs appreciated starting with physical brochures, followed by detailed explanations from trusted professionals. Midwives were seen as reliable sources who provided more thorough explanations. HCPs also recognized that midwives and obstetricians are best placed to deliver vaccination information due to their regular contact with women during pregnancy, while professionals such as youth healthcare physicians or paediatricians could support by addressing specific concerns, particularly around RSV-related illness. HCPs mentioned that group-based prenatal care, such as Centering Pregnancy, offers space for peer discussion without professional involvement. Regarding timing, both groups agreed that the second half of pregnancy is ideal for introducing information about neonatal immunization, as the postpartum period is often too overwhelming.

### 3.6. Influence

**Figure 1:**
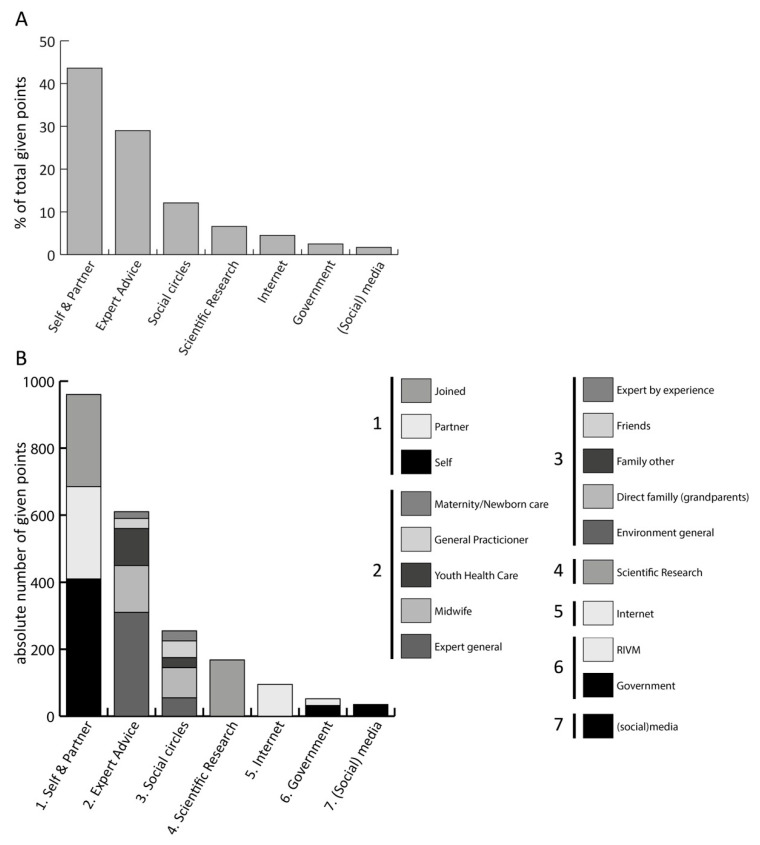
Factors influencing decision-making by (pregnant) women. All (pregnant) women (n = 21) were asked to assign 100 points among those who they believed had the greatest influence on their decision. (**A**) the percentage of the total points (2100) assigned to each category. (**B**) presents the absolute distribution of points among each category. Abbreviations: RIVM = Rijks Instituut voor Volksgezondheid en Milieu (National Institute for Public Health and the Environment.).

In addition to discussing information needs and sources, EMs were asked to reflect on the relative influence of different actors in their decision-making process. We asked participants to allocate 100 points to those who they believed had the greatest influence on their decision (Figure 1). Most prioritized themselves and their partner (960/2100 points, 46% of all points), as they made the final decision. This was followed by expert advice, in particular the midwife, youth healthcare and a general expert. Interestingly, social circle, in particular (grand)parents, was 3rd and scientific evidence was only regarded 4th in importance, with only 255/2100 (12%) points and 168/2100 (8%) points, respectively. Some participants explained that while they value scientific knowledge in principle, they rely on healthcare professionals to interpret and translate this information for them in a practical and trustworthy way. Scientific evidence was therefore perceived as indirect or less accessible, and less influential in the actual decision-making process compared to personal relationships and direct communication with professionals. The internet, government, and social media had little impact. While some trusted the RIVM, others preferred websites perceived as more neutral, though not always reliable or difficult to navigate. While social media was often mentioned, it was mainly linked to negative vaccine portrayals, lacking credibility and rarely influenced final decisions.

## 4. Discussion

This study explored the perspectives of (expectant) mothers (EMs) and healthcare professionals (HCPs) on RSV prevention through maternal vaccination and neonatal immunization. Our findings highlight varying levels of RSV awareness among EMs, broad professional support for prevention strategies, and a preference for integration within the National Immunization Program (NIP). To successfully implement a new RSV vaccination strategy, it is essential to understand and incorporate the perspectives of these stakeholders. Through semi-structured in-depth interviews with 21 EMs and 32 HCPs across The Netherlands, we explored the following key themes shaping attitudes and readiness: *RSV Awareness and Impact, RSV Protection & Vaccine Hesitancy, The Most Suitable Approach, Timing of Neonatal Immunization, Information and Influence.*

The majority of both EMs and HCPs expressed support for integrating RSV-protection into the NIP, with a clear preference for maternal vaccination. This overall acceptability is likely driven by increasing awareness of RSV and the perceived burden it places on both families and the healthcare system, as also recognized in previous literature [7,13]. The preference for maternal vaccination aligns with earlier studies highlighting maternal vaccination as a favorable strategy due to the immediate protection conferred after birth and avoiding of neonatal injections [7,10]. Many EMs, obstetricians and midwives compared RSV vaccination to pertussis ‘22-week shot’, which is already embedded in routine care and has an uptake of 70% in The Netherlands, suggesting that co-administration could facilitate implementation. The implementation of the pertussis vaccine in 2019 offers valuable lessons to enhance uptake even further, and various strategies have been discussed in literature. While approaches to RSV vaccination vary across Europe there is broad consensus that educational efforts improve uptake, and that HCPs remain the most trusted source of vaccine-related information [14]. A recent Canadian study also emphasized the importance of providing clear RSV information, as a notable proportion of parents lacked sufficient knowledge and confidence regarding the virus and its prevention [15]. Furthermore, keys to successful acceptance are a strong intention to vaccinate, defined as the readiness to accept vaccination, and the access to unbiased, comprehensive information [16]. In line with previous research, participants in this study emphasized the value of online decision aids, group-based prenatal care (e.g., Centering Pregnancy) and support by the midwife, particularly for those still undecided [17,18].

Nevertheless, concerns have been raised about safety of vaccination during pregnancy (EMs) and vaccination timing in relation to birth (EM/HCP). Notably, pregnant women who decide early against vaccination tend to maintain that decision throughout pregnancy. In addition, HCPs expressed concern about ensuring equal access to vaccination for all pregnant women. In the current Dutch healthcare system, pregnant women have regular appointments with midwives or obstetricians, while vaccinations are provided separately by Youth Health Care. As a result, even women intending to get vaccinated may encounter financial or practical barriers that prevent them from attending the extra appointment, leading to missed vaccinations. This represents an ongoing challenge that has yet to be effectively addressed within the existing system.

All EMs state that their child’s protection always outweighs their personal preference. This means that, although most EMs prefer maternal vaccination if the choice was offered, the majority will also accept neonatal immunization if this becomes the standard. Findings from our previously performed survey underscore this option, indicating that high uptake is achievable for both maternal and neonatal immunization strategies [7]. A major advantage of neonatal immunization is the high rate of protection independent of gestational age at birth and the option to adjust the vaccination schedule according to seasonal variation (EM/HCP). However, many interviewees mention concerns about vulnerability of the newborn (EM) and emotional resistance of new mothers in the first week post-partum influencing the willingness to accept vaccination of the newborn (EM/HCP). For this reason, the timing of the injection was a topic of discussion in many interviews with both EMs and HCPs.

For optimal protection of young infants, vaccination as early as possible is essential. Recent data from Spain demonstrate a high uptake (91.7%) of the anti-RSV monoclonal antibody Nirsevimab at the first day of life, with a corresponding reduction of 89.8% of RSV-related hospitalization [4]. However, EMs expressed hesitancy towards vaccination at this early time point, describing this period as an emotional and overwhelming time. They were more inclined to accept immunization if it was combined with another procedure, such as the heel prick screening at day 3–4 or the first scheduled vaccination. HCPs, in particular midwives, agreed with this view and expect that there is limited support for early neonatal immunization. The Netherlands differs from many other countries in that intramuscular injection immediately after birth, such as for Vitamin K, is not standard practice. This is largely due to the availability of an effective oral alternative, which has led to widespread acceptance of non-invasive administration routes. Given this context, the uptake of Nirsevimab administered directly postpartum is expected to be low in The Netherlands specifically. If Vitamin K injected is already integrated into standard care, uptake of RSV-immunization post-partum is expected to be higher [19]. However, midwives noted that, in their view, a comparable alternative exists in this case as well: maternal vaccination, which offers protection without requiring a neonatal injection.

To make a well-informed decision EMs need to receive the right information at the right time. They indicate that the final decision is made together with their partner, supported by information provided by an expert and in some cases their personal environment. Information needs to be presented in a clear and neutral manner, preferably in multiple languages and needs to be balanced when considering the severity of RSV. As decisions regarding vaccination during and after pregnancy typically begin during the antenatal period, it is essential for healthcare professionals, such as midwives and obstetricians, to foster a trusting and open relationship with expectant parents [20]. While healthcare providers acknowledge that discussing vaccine hesitancy can be challenging, particularly in maintaining an unbiased attitude, these conversations also present an opportunity to demonstrate empathy, address concerns respectfully, and strengthen the therapeutic relationship [20]. Given that expectant mothers view healthcare professionals as their most trusted source of information, these findings underscore the importance of equipping HCPs with the necessary tools, time, and training to effectively deliver tailored, accessible, and culturally sensitive communication.

Our study has several limitations. First, although more EMs were initially approached, a portion did not respond to emails or phone calls, while others declined participation due to time constraints or having recently given birth. Second, while EMs were interviewed, fathers were not included in the conversations but play a significant role in decision-making regarding the vaccination of their newborns. A third limitation is that we spoke with individual healthcare professionals rather than representatives of their professional organizations. As a result, the views and opinions may reflect personal perspectives rather than widely recognized or evidence-based positions held at the organizational level. Fourth, the participating EMs were specifically recruited from the uptake study, therefore all were in the second half of pregnancy or already gave birth. More importantly, while this allowed for in-depth exploration of their attitudes and decision-making processes, it may also have introduced a degree of response bias. By the time of the interviews, these women had already completed a questionnaire on RSV immunization, which may have prompted reflection and increased their baseline knowledge. As a result, their views could have been unconsciously influenced by prior exposure to information or the act of having considered the topic in advance.

## 5. Conclusions

Taken together, there is a strong willingness among EMs and HCPs to accept RSV prevention. While concerns remain regarding vaccine safety and the practical aspects of implementation, the perceived urgency of preventing RSV increases overall acceptance. Most HCPs highlight the importance of respecting the autonomy of EMs within the Dutch healthcare context. Offering a complementary strategy or offer women a choice both maternal and neonatal immunization options, is seen as the most effective strategy to achieve high uptake of RSV protection. EMs similarly value having the autonomy to choose the method of immunization that best aligns with their personal preferences and circumstances. Offering both strategies will give parents a choice, but also a second chance (YHCD1). It is essential that parents receive neutral and accessible information to support informed decision-making, with healthcare professionals playing a supportive role. Respecting parental autonomy is key, and this can be strengthened by offering a choice between different forms of RSV protection. Rather than pointing to a single priority intervention, our findings suggest that an effective public health strategy should consist of a coordinated package of measures, addressing communication, neutral and accessible information, service delivery, and decision-making support in a complete manner. Further research on the implementation strategies and their real-world effectiveness is essential to inform equitable, efficient, and sustainable RSV-prevention policies.

## Figures and Tables

**Table 1:** COREQ Summary Table: Key Methodological Elements.

COREQ Domain	Item	Study Details
Research Team and reflexivity	Interviewers	1st and 2nd author (LL and LH) with one of two research nurses (RN).
	Training and experience	Medical doctors and postdoctoral researchers at the Spaarne Hospital and one of two research nurses (working at the Spaarne Hospital).
	Relationship with participants	No prior relationship established
2. Study design	Participant groups	21 expectant mothers (EMs) and 32 healthcare professionals (HCPs)
	Recruitment strategy	EMs: random selection and approach of participants of previous Uptake study [7] HCPs: via networks, associations, and direct outreach; aiming for disciplinary and regional diversity
	Setting of interviews	Online
	Interview guide	Semi-structured topic guide, see data collection and Table S2
	Data saturation	Participant recruitment continued until thematic saturation was reached, which occurred after the inclusion of 21 EMs and 32 HCPs. This was informally assessed during data collection; jointly evaluated by the research team
3. Analysis and findings	Coding approach	Thematic analysis; coded by LL and LH; disagreements discussed and resolved
	Software used	MAXQDA Analytics Pro (version 24.4.1)
	Participant quotations included	Yes, representative, anonymized quotes included to illustrate key themes, Table 4

**Table 2:** Baseline characteristics (expectant) mothers.

**Baseline Characteristics (Expect) Mothers, *n* = 21**	
Age (Years)	31 [24–31]
Pregnant (n, %)	9 (42.8)
Gestational age (Weeks)	34 [30–37]
Recently given birth (n, %)	12 (57.1)
Newborn age (Weeks)	8.5 [3.8–10.0]
Multiparous women (n, %)	10 (47.6)
**Opinion in Previous UPTAKE-Questionnaire Study, *n* (%)**	
Maternal vaccination: Yes Neonatal immunization: Yes	9 (43)
Maternal vaccination: Doubt, likely yes Neonatal immunization: Doubt, likely yes	4 (19)
Maternal vaccination: Doubt, likely yes Neonatal immunization: Doubt, likely no	3 (14)
Maternal vaccination: Doubt, likely no Neonatal immunization: Doubt, likely no	2 (10)
Maternal vaccination: No Neonatal immunization: Doubt, likely no	2 (10)
Maternal vaccination: No Neonatal immunization: No	1 (5)

Numbers are expressed as *n* with percentages (%) or median with [Q1–Q3]. All women who were pregnant or had recently given birth completed a questionnaire exploring their opinions on RSV prevention measures previously [7]. The second section of this table addresses their willingness to accept maternal vaccination and neonatal immunization if offered, based on these questionnaires.

**Table 3:** Baseline characteristics of healthcare professionals.

Baseline Characteristics Healthcare Professionals, *n* = 32	
Profession	
General Practitioner	5 (16)
Pediatrician	5 (16)
Midwife	6 (19)
Obstetrician	5 (16)
Youth Healthcare Doctor	5 (16)
Youth Healthcare Nurse	6 (19)
Work area *	
North	2 (6)
East	5 (16)
Central	4 (13)
South	10 (31)
West	11 (34)
Population **	
Urban	9 (28)
Provincial	9 (28)
Mixed	14 (44)

Numbers are expressed as number with percentages (%). * For this interview, the work area was categorized by province as follows: North (Friesland, Groningen, Drenthe), East (Overijssel, Gelderland), Central (Utrecht, Flevoland), South (Limburg, North Brabant, Zeeland), and West (North Holland, South Holland). ** Population was categorized as follows: Urban (primarily patients residing in large cities), Provincial (primarily patients from rural areas or villages), and Mixed.

**Table 4:** Illustrative quotations.

Quotations per Theme from Pregnant Women and New Mothers (PNMs) and Healthcare Professionals (HCPs)
*RSV awareness and Impact*
PNMs
Well, I think part of it comes from your direct environment, so I also think that I haven’t worried too much about RSV so far because no one I know has experienced a severe case with their child. (INT010)
I have seen first-hand how quickly things can escalate, especially with a newborn, where it [RSV] can turn severe in an instant. (INT007)
When our child had RSV, they [doctors] didn’t know where there was [hospital] space due to so many RSV cases [in the country]. At one point, we thought we might have to travel far for a spot. Luckily, after eight hours of waiting, a bed became available nearby, which was a huge relief. But as soon as our child was off the Airvo [respiratory support], it was like, “Okay, you’re going home, the next patient is coming in.” (INT002)
For parents, having a child in the hospital is something that I think is often underestimated by others. Even though the chance of a child dying from it is small in The Netherlands, the impact on parents is huge. (INT019)
HCPs
RSV protection is such a no-brainer for me. But that’s easy to say from my role as a pediatrician, as I see all the sick children. I think people who are familiar with the situation won’t hesitate, but there are also many who don’t realize how seriously ill children can become—Ped5 (INT125)
I think as a general practitioner, you also regularly experience that in November, you can’t admit any child because you know the hospital is completely full of [children with] respiratory infections.—GP5 (INT120)
Every year, we [the hospital in general] have huge issues with RSV admissions, and this can even lead to admission stops in our gynaecology department. This, in turn, affects neonatology and our overall admission capacity, which means we are able to handle fewer patients. So, what I have encountered is that there is a real need for prevention due to these logistical challenges.—Obs4 (INT119)
I have many people in my practice who start asking themselves: Could they [my child] have the RS virus? They are very concerned about it.—GP2 (INT114)
*RSV protection & Vaccine Hesitancy*
PNMs
With all the knowledge and research we have now, it is amazing that we have come this far. I think we should consider ourselves fortunate that it [vaccines] exists, that it is offered to us, and that its value is widely recognized. (INT016)
Well, I think it is especially important to explain what kind of diseases these [in vaccines] are and what kind of impact they can have. Because nowadays, when I hear people talk, you know, ‘measles’ or whatever, they just think, ‘Well, that is not a big deal.’ I think, that is all fine, but that is because it is so rare now. These diseases are being really underestimated, and people only see the dangers of the vaccine, not the disease itself. (INT015)
The fuss around the COVID vaccinations made me more sceptical. Before that, I was less doubtful about vaccines. I got all my vaccinations as a child, so I just went along with it, it is just something you do. But during COVID, I also heard about strange side effects from vaccines. And now, it is about your baby, right? (INT006)
I think it is unfortunate that the vaccination rate is declining. On the other hand, I also think it might be a good thing if day-care centres are allowed to say, ‘If we have too many unvaccinated children, we can refuse them’. (INT009)
HCPs
I do think there is definitely support for it [RSV vaccination], because, you know, it always shows up in the local media here as well, that the hospital is once again struggling because of RSV admissions.—Obs4 (INT119)
I don’t think it is wise for healthcare providers to decide to do things a certain way, but the option should remain open for parents to make their own choices.—MW3 (INT111)
General doubt about vaccination has been somewhat fueled by the COVID pandemic. That is actually the largest group, and just at a time when everyone is starting to rethink vaccinations, we are introducing all these new vaccines.—Obs3 (INT112)
A combination of options would allow parents to make a choice, but it also gives them a second chance. That’s how I would see it.—YHCD1 (INT101)
*The Most Suitable Approach*
PNMs
This is really based on the fact that I want to give my child as few shots as possible while still providing full protection. Well, of course, you want what is best, right? If it is better to vaccinate your child, then that is the way to go. But if it is not necessary, then I’d rather take the shots myself. (INT012)
I think it [vaccinating a newborn] is just a bit emotional, it is simply not pleasant to give a shot to a very young baby, even though I know it is not truly traumatic. But if I can take it instead and it still provides the protection my baby needs, then I just feel like that is a little less invasive, I believe. (INT020)
I think, yes, the shot itself isn’t pleasant for the child. They might be bothered by it for a moment, but I believe they quickly forget about it afterward. (INT017)
If the vaccine is only effective for a certain period, then I’d probably choose to have it administered during the season when the virus is most present, when I feel my child needs that protection the most. (INT015)
HCPs
If, of course, there is the option of a vaccination for pregnant women, I think parents would be more likely to go for that than having their newborn receive an injection during the postpartum period.—MW2 (INT110)
I notice some resistance to vaccination during pregnancy, so I think, on a national level, you’d be more likely to get support for an immunization for neonates, to be included in the overall vaccination program.—Obs3 (INT112)
I think the easiest to implement would be to vaccinate the babies, and I say that because it is easier to adapt to changes in peaks, like we experienced a few years ago when we suddenly had a peak in the summer due to COVID.—Ped5 (INT125)
Because I also notice that people often think, *‘If I can protect the baby by doing this, and then the baby doesn’t even need to be vaccinated until later…’* That is often the turning point where I see people change their minds if they are still unsure.—MW4 (INT113)
*Timing of Neonatal Immunization*
PNMs
I don’t know if I’d be too eager in the first week. If it is a strong recommendation, I’d think, well, there must be a good reason for it. But if not, then I might hesitate as well. (INT008)
I think in those first days, you are already on a rollercoaster, just trying to process everything. The maternity nurse is still around, the midwife is checking in, and there is the newborn screening in the first week. If another thing were added on top of that, I think I would have found it quite overwhelming during my postpartum week. But then again, you just go through with it anyway. (INT006)
Maybe at the same time as the heel prick screening, since you are already dealing with procedures. The baby is crying anyway, but at least that is done at home. (INT010)
HCPs
I don’t think there is any woman who is particularly eager to have her child injected so soon after giving birth—MW3 (INT111)
We are not against the RSV vaccine or injection, but the timing and organization of its administration present a significant challenge.—YHCD5/YHCN6 (INT 130/131)
I can imagine that the role at the health center also include home visits in the second week, where they weigh and measure the baby. I can see that if it [neonatal immunization] becomes a standard procedure, like when we do a heel prick, a hearing test, and offer vaccination, there could be a lot of support for it. Especially if it is not framed as a vaccine but rather as a kind of medicine that prevents the child from getting sick.—GP5 (INT120)
*Information*
PNMs
In my opinion, we received little information [about vaccination during and after pregnancy]. But I think that was also partly because we always very quickly and firmly stated, ‘Yes, we’re just getting all the vaccinations’. (INT018)
Of course, it [information on vaccination] should be accessible through the government, but I would like more information. I want the option to dive deeper, not just feel like the benefits are being heavily pushed. You know, understanding both sides: what happens if you do it, but also what if you don’t? That would allow for a better choice. (INT004)
For the 22-week vaccination, I only received a leaflet from a midwife. She did say, ‘Make sure to read this carefully, it is very important.’ But it was because I asked more questions that I learned more about it. There are plenty of people who see a leaflet, think ‘Oh, okay, I’ll read it,’ and then eventually it ends up in the trash. (INT021)
I think a physical conversation would always work best for me, especially if it is with a midwife, a maternity care provider, or someone from the youth health clinic, someone I trust. (INT015)
I think part of it comes from your immediate surroundings. I haven’t really worried about RSV because no one in my environment has had serious issues with it with their child. (INT010)
HCPs
You really need to provide judgment-free counseling, of course. But we all know that is not something we always manage to do.—MW2 (INT102)
You are constantly engaging with each other [pregnant women and midwife], feeling each other out, and learning to communicate. So I think information about RSV protection is most easily shared within that bond of trust—MW3 (INT111)
Providing information through infographics has proven to be very useful.—Ped2 (INT106)
I think influencers have more impact than doctors nowadays, but I don’t rule out the possibility that doctors can also have an impact.—Ped5 (INT125)
Our goal is never to convince people. Our goal is to provide proper information.—YHCD2 (INT102)

Abbreviations: YHCD = Youth Health Care Doctor, YHCN = Youth Health Care Nurse, GP = General Practitioner, Obs = Obstetrician, MW = Midwife, Ped = Pediatrician, INT = Interview number. For the specific interview topics discussed, we refer to Table S2 Clarification of certain aspects of a citation can be found between brackets.

## Data Availability

The original contributions presented in this study are included in summarized form in the article/Supplementary Material. Further inquiries can be directed to the corresponding author.

## Supplementary Materials

The following supporting information can be downloaded at: https://www.mdpi.com/article/10.3390/vaccines13101051/s1, Table S1: Consolidated criteria for reporting qualitative studies (COREQ): 32-item checklist; Table S2: Interview topics & open-end questions.
