# Supplementary material for: Boosting RSV Immunization Uptake in The Netherlands: (Expectant) Mothers and Healthcare Professionals’ Insights on Different Strategies
Source: Vaccines (Basel). 2025 Oct 14;13(10):1051. doi: 10.3390/vaccines13101051 (PMC12567916; doi:10.3390/vaccines13101051)
Supplement: Supplementary file 1 [file vaccines-13-01051-s001.zip › vaccines-3893921-supplementary.pdf]

Supplementary Material

Table S1. Consolidated criteria for reporting qualitative studies (COREQ): 32-item checklist

No - Item	Guide question	Description
**Domain 1: Research team and reflexivity**		
Personal Characteristics		
1. Interviewer/facilitator	Which author/s conducted the interview or focus group?	*Method section -Data collection:* 1st and 2nd author (LL and LH) with one of two research nurses.
2. Credentials	What were the researcher's credentials?	1st author: MD - PhD<br>2nd author: MD - PhD<br>3rd author: MD - MSc<br>4th author: MD - PhD<br>5th author: MD - PhD<br>6th author: MD - PhD
3. Occupation	What was their occupation at the time of the study?	1st author: Paediatric resident<br>2nd author: Postdoctoral researcher<br>3rd author: Youth healthcare physician<br>4th author: Obstetrician-gynecologist<br>5th author: Professor and pediatrician infectious diseases<br>6th author: Pediatrician
4. Gender	Was the researcher male or female?	*Method section -Data collection:* All females.
5. Experience and training	What experience or training did the researcher have?	*Method section -Data collection:* MD-PhD or RN.
Relationship with participants		
6. Relationship established	Was a relationship established prior to study commencement?	*Method section – Study population and recruitment:* No.
7. Participant knowledge of the interviewer	What did the participants know about the researcher?	*Method section -Data collection: medical doctors/researchers and research nurses.*
8. Interviewer characteristics	What characteristics were reported about the interviewer/facilitator?	*Method section -Data collection:* Medical doctors and postdoctoral researchers at the Spaarne Hospital) and one of two research nurses (working at the Spaarne Hospital).
**Domain 2: Study Design**		
Theoretical framework		
9. Methodological orientation and Theory	What methodological orientation was stated to underpin the study?	*Methods:* A qualitative cross-sectional study with semi-structured interviews.<br>*Methods – Data analysis:* Thematic analysis.
Participant selection		
10. Sampling	How were participants selected?	*Methods – study population and recruitment:* criteria of study population was stated.
11. Method of approach	How were participants approached?	*Methods – study population and recruitment:* EMs by mail or phone and HCPs through professional networks, colleagues, and professional associations.

Table S1. *Continued*		
**No - Item**	**No - Item**	**No - Item**
12. Sample size	How many participants were in the study?	*Results:* 21 EMs and 32 HCPs.
13. Non-participation	How many people refused to participate or dropped out? Reasons?	*Results:* For EMs 14 in total. *Discussion:* For EMs a portion did not respond to emails or phone calls, while others declined participation due to time constraints or having recently given birth. The number of HCPs who declined or dropped out is unknown, as recruitment was facilitated through professional networks, colleagues, and professional associations, and non-responders were not systematically tracked (*Methods - study population and recruitment).*
Setting		
14. Setting of data collection	Where was the data collected?	*Methods – Data collection:* Conducted online.
15. Presence of non-participants	Was anyone else present besides the participants and researchers?	*Results:* individual interviews; except one interview was conducted jointly with two HCPs (a youth healthcare physician and a nurse).
16. Description of sample	What are the important characteristics of the sample?	*Results:* Table 1 and 2.
Data collection		
17. Interview guide	Were questions, prompts, guides provided by the authors? Was it pilot tested?	*Method – Data collection and Table S2.*
18. Repeat interviews	Were repeat interviews carried out? If yes, how many?	None, as it was not necessary.
19. Audio/visual recording	Did the research use audio or visual recording to collect the data?	*Methods – Data collection:* Both audio- and video recorded.
20. Field notes	Were field notes made during and/or after the interview or focus group?	*Methods – Data collection:* Field notes were taken to capture relevant observations.
21. Duration	What was the duration of the interviews or focus group?	*Results:* Median 25 minutes for EMs and 29 minutes for HCPs.
22. Data saturation	Was data saturation discussed?	*Method – Data collection:* Participant recruitment continued until thematic saturation was reached, which occurred after the inclusion of 21 EMs and 32 HCPs
23. Transcripts returned	Were transcripts returned to participants for comment and/or correction?	No, as the audio and video recordings provided sufficient clarity for accurate transcription and analysis.
**Domain 3: Analysis and findings**		
Data analysis		
24. Number of data coders	How many data coders coded the data?	*Methods – Data analysis:* Two (LL and LH).
25. Description of the coding tree	Did authors provide a description of the coding tree?	No.

Table S1. *Continued*		
**No - Item**	**No - Item**	**No - Item**
26. Derivation of themes	Were themes identified in advance or derived from the data?	Partly based on pre-defined topic lists (*Table S2)*, but ultimately derived from the data (*Results*).
27. Software	What software, if applicable, was used to manage the data?	*Methods – Data analysis:* MAXQDA Analytics Pro (version 24.4.1)
28. Participant checking	Did participants provide feedback on the findings?	No, this was not feasible given the large volume of findings, the number of participants, and the diversity of opinions across different participant groups.
Reporting		
29. Quotations presented	Were participant quotations presented to illustrate the themes / findings? Was each quotation identified?	*Results and Table 3*.
30. Data and findings consistent	Was there consistency between the data presented and the findings?	*Results and Table 3.*
31. Clarity of major themes	Were major themes clearly presented in the findings?	*Results and Table 3.*
32. Clarity of minor themes	Is there a description of diverse cases or discussion of minor themes?	*Results and Table 3.*

EM: (Expectant) Mothers; HCP: Healthcare professionals.

Table S2. Interview topics & open-end questions

(Expectant) Mothers
Introduction
Experience and first reaction to the UPTAKE questionnaire
Vaccination in general
Own thoughts and motivation about vaccination and the national immunization program (NIP)
Respiratory Syncytial Virus (RSV)
Knowledge, experience and if applicable impact of experience
RSV Protection
Knowledge and awareness of RSV protection methods
Decision-making regarding RSV protection – reasons, influencing factors, and any change in preference since questionnaire
Practical considerations – timing of immunization, due date, and timing and source of information
Decision-Making Support
Information and support needed to make a decision
Who makes the decision and who influences it
Health Care Professionals
Introduction
Professional background – specialization
Familiarity with the topic and exposure to the national discussion
Respiratory Syncytial Virus (RSV)
Clinical experience with RSV, including contact with sick infants
Interactions with concerned pregnant women or mothers, including questions about RSV protection
RSV protection: Knowledge and Attitudes
Awareness and understanding of RSV protection methods
Perceived acceptability of different protection methods and influencing factors (e.g., timing of birth, barriers, and facilitators to implementation)
RSV immunization (the Health Council Recommendation in the Netherlands)
Support or opposition to the recommendation, including motivations and concerns
Practical considerations for implementation (e.g., timing of the shot, especially in newborns)
Roles and responsibilities in providing information and administering the immunization
Decision-Making Support
Which support do (expectant) mothers need to make informed decisions
Who should provide this information
Who does influence the decision and who does ultimately make the decision
General views on vaccination
Perspectives on the declining vaccination rates in the Netherlands and how this trend may affect the acceptance and implementation of new vaccines

RSV: Respiratory Syncytial Virus
